# Knowledge, Attitude and Practices of Sugar-Sweetened Beverages: A Cross-Sectional Study among Adolescents in Selangor, Malaysia

**DOI:** 10.3390/nu12123617

**Published:** 2020-11-25

**Authors:** Nur Islami Mohd Fahmi Teng, Norsham Juliana, Nur Liyana Izlin, Nur Zulaikha Semaon

**Affiliations:** 1 Faculty of Health Sciences, Universiti Teknologi MARA, Cawangan Selangor, Puncak Alam 42300, Malaysia; liyanaizlin18@gmail.com (N.L.I.); eykasemaon97@gmail.com (N.Z.S.); 2 Faculty of Medicine and Health Sciences, Universiti Sains Islam Malaysia, Kuala Lumpur 71800, Malaysia; njuliana@usim.edu.my

**Keywords:** sugar-sweetened beverages, body composition, adolescents

## Abstract

This study aims to examine the level of knowledge, attitude and practices (KAP) of adolescents towards sugar-sweetened beverages (SSB), together with the associated factors that determine their KAP. Data were collected using self-administered questionnaires that consisted of sociodemographic, the KAP for the SSB questionnaire, and the Beverage Intake Questionnaire (BEVQ). The respondents’ heights, weights, waist circumferences and body fat percentages were measured. This study involved 439 adolescents aged between 13 and 17 years old, in public secondary schools in Selangor, Malaysia. The results reveal that 35% of the adolescents were overweight, 26% had a high waist circumference, and 45% had a high body fat percentage. Caffeinated drinks and full cream milk were the most frequently consumed SSBs. The KAP score revealed a good attitude (88.4%), a moderate knowledge (51.8%) and a poor practice (40.5%). Those with a higher body fat percentage showed significantly good attitude scores (*p* < 0.05). Low household income groups, females, adolescents aged 16–17 years old and being from an urban area demonstrated a significant (*p* < 0.05) positive determinant towards the KAP score. In conclusion, high awareness of negative health outcomes associated with SSBs among adolescents was not in accordance with the level of their lifestyle choices.

## 1. Introduction

Sugar-sweetened beverages (SSBs) are defined as beverages with added sugar, and include sports drinks, sugar-sweetened tea and coffee, soft drinks and electrolyte-replacement drinks. The calories contained in SSBs provide little to no nutritional value and less satiety, as compared to solid food. Hence, SSB intake leads to unhealthy weight gain resulting from a high total energy intake, with little nutritional value [1]. Globally, SSB intake is highest among adolescents aged between 13 and 20 years [2]. The increase of the prevalence and degree of obesity amongst adolescents in many populations typically leads to significant public health problems. In Malaysia, the prevalence of obesity has increased from 5.7% in 2011, to 11.9% in 2015 [3]. A higher prevalence of obesity in Malaysia was found in urban areas, as compared to rural areas [4]. In Metropolitan Kuala Lumpur, the prevalence of overweight and obesity cases is even more worrying, with 34.2% of obesity cases being amongst young adults [5]. The high availability of SSBs, the high density of fast food chain outlets, and poor lifestyle choices, were amongst the factors that contributed to the country’s rising problems, such as diabetes, obesity and other non-communicable diseases [6]. In addition, a higher consumption of SSBs was associated with poor oral health, the development of metabolic syndromes, as well as cardio-metabolic risk factors such as type 2 diabetes, later in life [7]. The development of type-2 diabetes and excessive weight gain was reported to be strongly related to the consumption of added sugar [8]. 

Great effort has been made to alert the population about the hazardous effects of high sugar intake as a major contributing factor to the rise of obesity prevalence and metabolic diseases. However, a report by Amarra et al. (2016) highlighted that the daily intake among Malaysian adolescents was still above that recommended by the WHO [9]. MyHeARTs study in 2016 reported that adolescents in Malaysia were highly susceptible to a high intake of sugar, with the disparity of intakes being based on geographical locations of the adolescents [10]. Pengpid and Peltzer (2020) mentioned that adolescents’ intake of SSB, specifically in terms of carbonated soft drinks, is not just associated with metabolic problems, but also with poor mental health [11]. The majority of the consumption of daily added sugar by adolescents in Malaysia involved SSBs. They were reported to consume beverages at any time of the day, with the most frequent consumption during breakfast [11]. 

To date, data in the area of knowledge, attitude and practices (KAP) of SSB are still lacking. In Malaysia, studies on SSB are mostly limited to the trend of intake. It is critical to comprehend in-depth whether the adolescents have sufficient knowledge on SSBs in order to appraise their choice and rate of SSB intake. Theoretically, the KAP questionnaire is able to reveal any misconceptions or potential barriers to behavior change. Thus, the information further leads to effective intervention strategies that integrate the influence of cultural factors. Hence, the primary objectives of this study are to determine the level of KAP on SSBs and the socio-demographic determinants amongst adolescents in secondary schools in Selangor, Malaysia. This study also intends to describe the beverage intake and the anthropometric measurement among adolescents. The findings of this study will serve as a baseline for developing appropriate intervention measures and raise awareness on the negative impact of SBBs among adolescents. 

## 2. Materials and Methods 

A cross-sectional study was conducted across three secondary schools in Selangor. Selangor is the most developed state in Malaysia, and is divided into nine districts. It is located at the west side of the country. The total population is around 6.53 million, which includes three main ethnicities, namely Malay, Chinese and Indian, with Malay being the majority [12]. This study included male and female participants aged between 13 and 17 years. In the Malaysian education system, adolescents in this age range are generally students in secondary school. Participants who had diseases, were on medication, or fall under the ‘special needs’ category were excluded from the study. A sample size of at least 385 adolescents was determined using Krejcie and Morgan’s equation from a population of 322,164 registered secondary school students in Selangor, with a 95% confidence interval. Data collection was performed and completed in November 2019.

This study involved a convenience sampling design. A complete list of 261 secondary schools in Selangor was obtained from the Department of Education of Selangor. A total of three schools, each from a different district, were selected. The selection was performed using a multi-staged cluster sampling design. The school with the greatest number of students in each selected district was chosen. Subsequently, all students who fulfilled the inclusion criteria were invited to participate in the study. The consent form was handed to the participants one day before the data collection was carried out. Participants consisted of students who attended school on the day of the data collection, and had handed in the signed-off consent form. A total of 450 consented forms were received.

A set of validated questionnaires was administered to the adolescents. The questionnaire consisted of socio-demographic sections, KAP questionnaires [13], and a Beverage Intake Questionnaire (BEVQ), which were adopted from Hedrick et al. (2012) [14]. 

A predefined self-report questionnaire was adopted to identify the KAPs for SSBs among adolescents. It involves a multistep process that comprises both reporting, as well as the cultural adaptation of these questionnaires. This questionnaire has been validated among young adults population in Malaysia [13]. The KAP questionnaire consists of 20 dichotomous questions (10 items for knowledge, 5 items for attitude and 5 items for practice) that require a response of either Yes/No or Agree/Disagree. This questionnaire has been tested for reliability and validity in both the English and Malay languages [13]. The overall score was calculated by summing the correct responses by the participants. The maximum score for each domain is equal to the number of items, therefore the higher score reflects a higher (good) indicator. Furthermore, each domain was categorised into two levels of indicators, ‘poor’ or ‘good’, based on the median of results. In the present study, ‘poor knowledge’ was indicated by a score of ≤4, while ‘good knowledge’ by a score of ≥5. Similarly, ‘poor attitude’ was indicated by a score of ≤4, while ‘good attitude’ by a score of 5, and ‘poor practice’ was indicated by a score of ≤1, while ‘good practice’ by a score of 2. 

The adopted BEVQ investigates the frequency of plain water (bottled water/tap water) and SSB intake on a weekly basis [15]. It consists of 10 types of beverages, including plain water, 100% fruit juice, sweetened fruit beverages, full-cream milk, low-fat milk, skimmed milk, regular soft drinks, energy and sports drinks, and alcoholic drinks. This questionnaire estimates the habitual intake of these beverages, which are part of the measurement of practices. The respondents were requested to indicate “how often” they consumed a beverage in the past month. There are seven categories of responses, ranging from “never or less than 1 time per week”, to “more than two times per day”. However, during analyses, we further simplified the categorisation into three primary categories, namely, “never or less than 1 time per week”, “6 times per week” and “daily (1 time or more per day)”.

Anthropometric measurements such as body weight, height, waist circumference and body fat percentage were measured by a trained researcher. Body weight was measured in light clothing, without shoes, using a portable digital electronic scale (Seca 813 digital electronic weighing scale). Height was measured as the distance from the top of the head to the bottom of the feet, without shoes, using a calibrated vertical stadiometer (Seca Portable 217). The BMI was calculated as kilogram body weight/m^2^. The waist circumference was measured at the narrowest part, between the lower rib and the iliac crest, using a non-elastic flexible tape, and recorded to the nearest 0.1 cm. The percentage of body fat was assessed using a bioelectrical impedance, which consisted of a bipolar handheld device (Omron HBF-306, Omron, Japan), and the results were recorded as percentage of body weight. The subjects stood with their feet slightly separated, holding the device in both hands, arms stretched out at an angle of 90 degrees relative to the body, while the instrument recorded the impedance from hand to hand, and subsequently calculated the percentage of body fat to the nearest 0.1%, based on age, gender, height and weight [16].

The classification of BMI was based on the international obesity task force (IOTF) criteria. This criteria was used because it was shown to be most appropriate amongst the Asian adolescent population [17]. Central obesity was defined by the waist circumference’s ≥90th percentile, according to the waist circumference percentile curves for Malaysian children and adolescents. The cut-off value used was 83.8 cm for males, and 78.8 cm for females [18]. The cut-off points for the normal body fat percentage was less than 30% for boys, and less than 35% for girls. This cut-off value is based on the body composition assessment for the development study of an international growth standard for pre-adolescent and adolescent children [19].

The Statistical Package for Social Science (SPSS) version 21.0 was used for tabulation and analysis of the collected data. Scatter plots, skewness, and kurtosis were examined to determine the normality of the data distribution. Once the data distribution was determined, the not normally distributed median was then taken. The demographic data was examined using descriptive statistics, and the results were reported as frequency and percentage values. The knowledge, attitude and practice (KAP) scores were calculated by summing up the participant’s number of correct responses. Mean percentage score was calculated by dividing the mean score with maximum score and multiplying it by 100. An independent *t*-test was used to determine any significant difference between genders with regard to the KAP scores.

The frequency of plain water and SSB consumption were described via descriptive statistics, and the results were reported as a frequency and percentage. Chi-square analysis was also performed to test relationships between genders and the frequency of consumption.

The binary logistic regression analysis was carried out to verify the independent effects of the predictors on the dependent variables and predictors with a *p*-value ≤ 0.25, which was entered in the multivariable logistic regression analysis model to identify the final predictors of KAPs, after controlling other independent variables. The odds ratio and 95% CI were calculated, and the result (*p* ≤ 0.05) was considered statistically significant. The result was described in text form, before being summarized and presented in tables. 

All subjects gave their informed consent for inclusion before they participated in the study. The study was conducted in accordance with the Declaration of Helsinki, and the protocol was approved by the Ethics Committee of Universiti Teknologi MARA (REC/123/17).

## 3. Results

### 3.1. Socio-Demographic Characteristics and Anthropometric Profile of Participants

The socio-demographic characteristics of the participants are shown in Table 1. A total of 439 out of 450 respondents completed all of the questionnaires and measurements. The response rate was 97.6%. The prevalence of being overweight was 18.5% (*n* = 81), while obesity was 16.9% (*n* = 74). The majority of the respondents (73.8%, *n* = 324) had a normal waist circumference level. However, slightly more than half of the respondents (55.4%, *n* = 243) had a normal body fat percentage.

### 3.2. Mean Score of Knowledge, Attitude and Practice on SSB among Participants

The KAP score of the participants towards SSB intake was calculated separately, and presented in Table 2. Each correct response represents a score of 1, and each wrong response represents a score of 0. Therefore, higher scores reflect better results. The highest mean percentage score was 88.43% for attitude, followed by a mean percentage score of 51.84% for knowledge, and the lowest mean score of 44.56% was for practice. This result indicates a good (positive) level of attitude, moderate level of knowledge and poor (negative) level of practice. There is a significant difference (*p* < 0.001) between gender, where females revealed higher attitude scores compared to males. 

### 3.3. The Frequency of Beverage Intake of Participants

Table 3 shows the frequency of beverage intake by the participants. On a daily basis, plain water (84.3%) was the most common beverage intake, followed by sugar-sweetened coffee or tea (25.5%) and full cream milk (16.6%). Thus, we can conclude that, among the participants, at least one-quarter of them consumed SSB on a daily basis. More than half (60%) of the participants consumed SSB less than once a week. Alcoholic drinks had the lowest frequency intake, with 98.9% of the participants consuming such beverages either once a week, or not at all. This low intake may be due to religious and age reasons, since the majority of the participants were Muslims and under-age. Further analyses revealed that trends of fruit juice, sweetened fruit beverage, low fat milk, skimmed milk, regular soft drink, sugar-sweetened coffee or tea, energy drink and sport drinks and others consumption are highly associated with gender preference. 

### 3.4. Factors Associated with Participants’ Knowledge Level on SSB

Amongst the variables entered in the bivariate analysis, only family monthly income has shown a significant association (Table 4). A family monthly income within the range of RM 1501–RM 8000 was more likely to get a good score for knowledge (score of ≥5), with the best score in the range of family monthly income being RM 1501–RM 2500 (crude OR = 0.50; 95% CI = 0.26–0.98). On the other hand, variables with a *p*-value ≤0.25, which were entered in the multivariable logistic analysis, and some of the above associations, did not exist after adjustment for other variables. In the multivariable logistic analysis, subjects whose families earned an average monthly income of RM 1501–RM 2500 were 0.51 times (adjusted OR = 0.51; 95% CI = 0.26–0.99) and 0.45 times (RM 2501–RM 5000, adjusted OR = 0.45; 95% CI = 0.22–0.92) more likely to have an excellent knowledge on SSB, as compared to those who came from families who earned ≥RM 5000 a month.

### 3.5. Factors Associated with Participant’s Attitude Level on SSB

In the bivariate analysis, two factors were significantly associated with a good attitude score (score = 5) for SSB intake, which were the age category and type of school area, as shown in Table 5. In the attitude domain, participants aged between 16 to 17 years old showed a good attitude (crude OR = 0.5; 95% CI = 0.27–0.94), as compared to younger participants (13–15 years old). Participants at urban schools had the best score in the attitude level for SSB intake (crude OR = 1.71; 95% CI = 1.16–2.54), as compared to those in rural schools. In the multivariable logistic regression analysis, female participants had a score of 0.41 (adjusted OR = 0.41, 95% CI = 0.27–0.62) and participants aged between 16 to 17 years old had a score of 0.44 (adjusted OR = 0.44, 95% CI = 0.22–0.85), which were more likely to get a good attitude level for SSB. Participants from urban schools were 1.78 times (adjusted OR = 1.78, 95% CI = 1.16–2.72) more likely to have a good attitude on SSB intake compared to participants from rural schools. In addition, participants with parents who were in non-professional occupations (adjusted OR = 1.85; 95% CI = 1.10–3.14) were more likely to have a good attitude toward SSB intake, compared to the professional.

### 3.6. Factors Associated with Participant’s Practice Level on SSB

With regard to the practice score, there were no associations between the socio-demographic characteristics in the bivariate logistic regression analysis, as shown in Table 6.

### 3.7. The Association between KAP on SSB with Body Composition

Table 7 shows the association between KAPs on SSB intake, with regards to body composition. No association was found between KAPs on SSB intake, with that of body mass index, and waist circumference level. However, there was a significant association (*p* < 0.05) found between KAP on SSB intake, and body fat percentage. The participants who had a high body fat percentage had a much higher (good) attitude percentage score.

## 4. Discussion

The findings revealed a good level of attitude, moderate level of knowledge, and poor level of lifestyle choices amongst SSB intake of the participants. The good attitudes show that the participants were more attentive toward reducing the SSB consumption. In general, they were aware of the needs to read the food labels and ingredients, and to choose beverages without simple sugars. This shows that, although they had a good attitude towards SSB intake, it did not change the way they limited this intake. This is in agreement with prior work [20], which suggests that these positive attitudes are neither preventive, nor health-promoting. As the level of knowledge was considered relatively low, this may reflect the low level of awareness amongst the participants in practicing good habits associated with avoiding SSBs and limiting their intake in general.

The findings of this study pointed out that the most frequently consumed beverage on a daily basis was plain water. This finding is similar to that in the study by Zahran [21], in which most of their participants consumed plain water on a daily basis. Tea and caffeinated drinks (25.5%) were the most consumed SSBs on a daily basis amongst the participants in the present study. This trend is similar to studies by Norimah et al. [22] and Teng et al. [15], which reported a high consumption of coffee and tea amongst their participants. These findings are in agreement with a study that demonstrated that the reasons behind the high consumption of coffee and tea amongst students included helping them to concentrate, keep them awake at night during exam preparation phases, and staying active during classes [23]. The majority of the participants either never drank SSB, or consumed it less than once a week. This reflects that this particular population sample consumed SBBs occasionally rather than regularly. Alcoholic drinks were the least consumed beverages. This is because of religious issues, since most of the participants were Muslims, and all Muslims are prohibited from consuming alcoholic drinks. Our result also indicated that the trend of intake for different type of beverages are associated with gender. This is in accordance with the results for their KAP, which highlighted the significant difference in attitude domain between genders. 

There was an association between knowledge levels on SSBs, and monthly family income of the participants. It was reported that low level family monthly income groups were a contributing factor toward having excellent knowledge on SSB, as compared to those who earned ≥RM 5000 as a monthly family income. Income-related factors have previously been described as factors that are attributed to inequalities of knowledge in nutrition [24]. This study brought forward interesting findings that demonstrate that those with low income also reflected good knowledge in SSBs. This finding is in agreement with the suggestion by Xu et al. (2020), who mention that the inequality of knowledge is also strongly related to geographical areas with good accessibility to education facilities [25]. Hence, the area considered in this study has good accessibility to facilities, such as well-equipped public educational institutions and multiple areas with free internet hot spots. All these knowledge related sources can be gained by anyone, without any additional cost. However, another important issue that needs to be addressed is that this knowledge cannot be translated into practice by those families with limited income, as they do not have the option to buy healthier foods such as fresh fruits and vegetables. The concern was more on satisfying hunger, and to fulfil the desire of food with good taste. Therefore, there was a higher tendency to buy high sugar foods and SSBs [26]. It was also shown that parental influence on the intake of a child’s drinks may be linked to the knowledge associated with diet and health [27]. Based on previous studies for children and adolescents, it was shown that the availability of SSBs was correlated with the child’s intake, which was much better correlated amongst well-educated and high income parents [28]. The participants who had a high income could explain that they afforded to buy SSBs, which led to their children consuming more SSBs, as compared to low income level groups. As in agreement with a recent systematic review [29], we also found that neither level of education nor income have any association with good practices in SSBs intake. 

This study discovered that gender, age and location of school impact the overall attitude towards SSB intake. Females of older age, living in urban areas were found to have a good awareness (attitude) towards SSB intake. Similar observations have also been reported in previous studies amongst Australians [30]. Our findings also support previous studies showing that boys had a higher intake of SSBs than girls [31]. Females may respond more favourably to these efforts, as they appear to be more concerned about body weight issues, and are more likely to rate behaviours of food choice as an essential means for their life, as compared to males [32]. The same goes to the participants who were from urban schools, as they had a good attitude, compared to those who were from the rural schools. This is because rural residents have many characteristics, including widespread socioeconomic disadvantages, and can easily access unhealthy foods that increase their risk for chronic diseases, food insecurity, poor dietary behaviours, and higher intake of SSBs [33]. 

The results of this study also demonstrate that 19% of the participants were overweight, and 17% were obese. This shows that one-third of the participants fall between the overweight and obese categories, which reflects the high rate of overweight and obesity issues amongst the adolescents. These findings were almost similar to those from a related study that was also conducted amongst adolescents in Malaysia [34]. The findings of this study also found that the average waist circumference measurements of the respondents were 74 cm for boys, and 75 cm for girls. In a study conducted by Higgins et al. [35], it was stated that children with a waist circumference level of 71 cm or more, were 14 times more likely to develop a negative risk profile than a normal one. Therefore, 26% of the boys and girls in this study had a high waist circumference measurement, and were more likely to develop a negative risk profile. The findings also show that nearly half of the participants had a high body fat percentage, which is associated with a high risk of diseases such as hypertension, type 2 diabetes, cardiovascular diseases and metabolic syndromes [36]. 

There were a few limitations in this study. Firstly, this study only involved small sample of schools, therefore the data could not represent the Malaysian adolescents. Next, the data for beverages intake in the survey were self-reported. Self-reported data has the potential of being underreported in terms of beverage consumption, especially for overweight or obese children, due to social desirability bias and other factors. While exploring the associations between SSB and body composition, other dietary, physical, genetic and environmental factors that could also impact the value of the body composition were not studied. Future studies investigating SSB consumption in Malaysia should also consider additional categories, such as low and no-calorie soft drinks and discretionary milk beverages such as bubble tea. 

Nevertheless, our findings have implications for future research. As we found a reasonable gap between knowledge, attitude and practices (KAPs), it is necessary to develop or strengthen strategies by which positive attitudes can be converted into promising practices. The education towards the improvement of knowledge of SSBs should be enhanced in the education syllabus. It is recommended to implement this strategy by taking into consideration the socioeconomic status, such as emphasizing on the nutrition information about SSBs as well as healthy eating among participants belonging to high income families. Attempts should also be made to reinforce the consequences of high SSB intake, in order to transform them to positive practices. The availability of SSBs should be minimised, both in school and at home, to support healthier lifestyle changes.

## 5. Conclusions

This study examined the associated factors that determine the knowledge, attitude and practices (KAPs) of adolescents towards SSBs. The results indicate that, amongst adolescents in secondary schools across Selangor, there was a good level of attitude and moderate level of knowledge, but a poor level of lifestyle choices towards sugar-sweetened beverages (SSBs). A good attitude was mostly observed amongst females and those with a high body fat percentage. 

The interventions associated with increasing knowledge and awareness towards the consequences of SSB intake should be carried out. The strategy of those interventions must include strategies to transform the knowledge received into healthier lifestyle changes by limiting the consumption of SBBs by adolescents. A different approach to the gender differences is suggested for a promising outcome. 

## Figures and Tables

**Table 1:** Characteristics of respondents (*n* = 439).

Socio-Demographic Characteristics	Frequency (*n*)	Percentage (%)
Gender		
Male	218	49.7
Female	221	50.3
Age		
13–15 years old	381	86.8
16–17 years old	58	13.2
Ethnicity		
Malay	422	96.1
Others	17	3.9
Type of school		
Rural	242	55.1
Urban	197	44.9
Father’s occupation		
Professional	211	48.1
Non-professional	228	51.9
Mother’s occupation		
Professional	150	34.2
Non-professional	289	65.8
Family monthly income		
≤RM 1500	52	11.8
RM 1501–RM 2500	117	26.7
RM 2501–RM 5000	131	29.8
RM 5001–RM 8000	72	16.4
RM 8001–RM 15,000	47	10.7
≥PM 15,000	20	4.6
BMI-for-age classification		
Underweight	39	8.88
Normal	245	55.81
Overweight	81	18.45
Obese	74	16.86
Waist circumference (cm)		
Normal	324	73.8
High	115	26.2
Body fat percentage (%)		
Normal	243	55.36
High	196	44.65

**Table 2:** The score of knowledge, attitude and practice on sugar-sweetened beverages (SSB).

Variable	Mean ± SD (Percentage)	Gender Mean ± SD		*p* Value
		Male	Female	
Knowledge Score	5.18 ± 1.57 (51.84)	5.19 ± 1.58	5.17 ± 1.58	0.915
Attitude Score	4.42 ± 0.91 (88.43)	4.22 ± 0.07	4.61 ± 0.73	0.000 **
Practice Score	2.22 ± 1.14 (44.56)	2.24 ± 1.20	2.22 ± 1.07	0.910

Maximum score: Knowledge: 10, Attitude: 5, Practice: 5. ** Significant level of *p* < 0.001.

**Table 3:** Frequency of beverages consumption (*n* = 439).

Types of Beverages	Never or ≤1 Time per Week *n* (%)	≤6 Times per Week *n* (%)	Daily *n* (%)	*p* Value (*X*^2^ Test)
Plain water	14 (3.2)	55 (12.5)	370 (84.3)	
Male	10 (2.3)	28 (6.4)	180 (41.0)	0.242
Female	4 (0.9)	27 (6.2)	190 (43.3)	
100% Fruit Juice	287 (65.4)	118 (26.9)	34 (7.7)	
Male	121 (27.6)	78 (17.8)	19 (4.3)	0.000 **
Female	166 (37.8)	40 (9.1)	15 (3.4)	
Sweetened Fruit Beverage	246 (56.1)	149 (33.9)	44 (10.0)	
Male	104 (23.7)	87 (19.8)	27 (6.2)	0.002 **
Female	142 (32.3)	62 (14.1)	17 (3.9)	
Full cream milk	205 (46.7)	161 (36.7)	73 (16.6)	
Male	93 (21.2)	85 (19.4)	40 (9.1)	0.233
Female	112 (25.5)	76 (17.3)	33 (7.5)	
Low Fat Milk (2%)	271 (61.7)	113 (25.8)	55 (12.5)	
Male	120 (27.3)	65 (14.8)	33 (7.5)	0.016 *
Female	151 (34.4)	48 (10.9)	22 (5.0)	
Skimmed milk	342 (77.9)	59 (13.5)	38 (8.7)	
Male	152 (34.6)	36 (8.2)	30 (6.8)	0.000 **
Female	190 (43.3)	23 (5.2)	8 (1.8)	
Regular Soft Drinks	287 (65.4)	118 (26.9)	34 (7.7)	
Male	127 (28.9)	65 (14.8)	26 (5.9)	0.001 **
Female	160 (36.4)	53 (12.1)	8 (1.8)	
Sugar-Sweetened coffee or tea	117 (26.7)	210 (47.8)	112 (25.5)	
Male	47 (10.7)	114 (26.0)	57 (13.0)	0.048 *
Female	70 (15.9)	96 (21.9)	55 (12.5)	
Energy and Sports Drinks	277 (63.1)	115 (26.2)	47 (10.7)	
Male	113 (25.7)	70 (15.9)	35 (8.0)	0.000 **
Female	164 (37.4)	45 (10.3)	12 (2.7)	
Alcoholic drinks	434 (98.9)	2 (0.5)	3 (0.7)	
Male	215 (49.0)	1 (0.2)	2 (0.5)	0.84
Female	219 (49.9)	1 (0.2)	1 (0.2)	
Other beverages	419 (95.4)	15 (3.4)	10	
Male	208 (47.4)	5 (1.1)	5 (1.1)	0.038 *
Female	219 (49.9)	2 (0.5)	0 (0)	

* Significant level of *p* < 0.05, ** Significant level of *p* < 0.01.

**Table 4:** Bivariable and multivariable logistic regression predicting knowledge on SSB among participants, (*N* = 439).

	*N* (%) with Score ≥ 5	Crude OR (95% CI)	*p*-Value	Adjusted OR	*p*-Value
				(95% CI)	
Gender					
Male	148 (67.9)	1.00		1.00	
Female	146 (66.1)	1.09 (0.73–1.62)	0.684	1.16 (0.77–1.74)	0.483
Age					
13–15	250 (65.6)	1.00		1.00	
16–17	44 (75.9)	0.61 (0.32–1.15)	0.125	0.66 (0.34–1.29)	0.224
Ethnicity					
Malay	284 (67.3)	0.69 (0.26–1.86)	0.469	0.7 (0.27–2.02)	0.548
Others	10 (58.8)	1.00		1.00	
Type of school area					
Urban	134 (68.0)	1.00		1.00	
Rural	160 (66.1)	1.09 (0.73–1.63)	0.673	1.14 (0.75–1.73)	0.553
Father’s occupation					
Professional	144 (68.2)	1.00		1.00	
Non-professional	150 (65.8)	1.12 (0.75–1.67)	0.585	1.02 (0.60–1.71)	0.955
Mother’s occupation					
Professional	106 (70.7)	1.00		1.00	
Non-professional	188 (65.1)	1.29 (0.85–1.98)	0.236	1.21 (0.74–1.97)	0.457
Family monthly income					
≤RM 1500	27 (51.9)	1.00		1.00	
RM 1501–RM 2500	80 (68.4)	0.50 (0.26–0.98)	0.042 *	0.51 (0.26–0.99)	0.048 *
RM 2501–RM 5000	94 (71.8)	0.43 (0.22–0.83)	0.012 *	0.45 (0.22–0.92)	0.029 *
RM 5001–RM 8000	51 (70.8)	0.45 (0.21–0.94)	0.033 *	0.51 (0.21–1.23)	0.135
RM 8001–RM 15,000	32 (68.1)	0.51 (0.22–1.15)	0.104	0.56 (0.21–1.45)	0.23
≥PM 15,000	10 (50.0)	1.08 (0.39–3.03)	0.884	1.17 (0.38–3.64)	1.167

* Significant level of *p* < 0.05.

**Table 5:** Bivariable and multivariable logistic regression predicting attitude on SSB among participants, (*N* = 439).

	*N* (%) with Score = 5	Crude OR	*p*-Value	Adjusted OR	*p*-Value
		(95% CI)		(95% CI)	
Gender					
Male	111 (50.9)	1.00		1.00	
Female	157 (71.0)	0.55 (0.21–1.46)	0.234	0.41 (0.27–0.62)	0.000 ***
Age					
13–15	225 (59.1)	1.00			
16–17	43 (74.1)	0.50 (0.27–0.94)	0.030 *	0.44 (0.22–0.85)	0.015 *
Ethnicity					
Malay	260 (61.6)	0.55 (0.21–1.46)	0.234	0.54 (0.19–1.51)	0.236
Others	8 (47.1)	1.00			
Type of school area					
Urban	134 (68.0)	1.71 (1.16–2.54)	0.007 **	1.78 (1.16–2.72)	0.008 **
Rural	134 (55.4)	1.00		1.00	
Father’s occupation					
Professional	133 (63.0)	1.00		1.00	
Non-professional	135 (59.2)	1.18 (0.80–1.73)	0.412	1.85 (1.10–3.14)	0.022 *
Mother’s occupation					
Professional	91 (60.7)	1.00		1.00	
Non-professional	135 (59.2)	0.98 (0.65–1.46)	0.906	1.08 (0.66–1.75)	0.759
Family monthly income					
≤RM 1500	31 (59.6)	1.00		1.00	
RM 1501–RM 2500	74 (63.2)	0.86 (0.44–1.68)	0.653	0.96 (0.47–1.93)	0.904
RM 2501–RM 5000	87 (66.4)	0.75 (0.39–1.45)	0.387	0.98 (0.46–2.06)	0.952
RM 5001–RM 8000	38 (52.8)	1.32 (0.64–2.72)	0.45	2.28 (0.93–5.57)	0.072
RM 8001–RM 15,000	29 (61.7)	0.92 (0.41–2.06)	0.832	1.42 (0.54–3.78)	0.48
≥RM 15,000	9 (45.0)	1.80 (0.64–5.11)	0.266	1.96 (0.61–6.32)	0.258

* Significant level of *p* < 0.05, ** Significant level of *p* < 0.01, *** Significant level of *p* < 0.001.

**Table 6:** Bivariable and multivariable logistic regression predicting practice on SSB among participants, (*N* = 439).

	*N* (%) with Score ≥ 2	Crude OR	*p*-Value	Adjusted OR	*p*-Value
		(95% CI)		(95% CI)	
Gender					
Male	153 (70.2)	1.00		1.00	
Female	168 (76.0)	0.74 (0.49–1.13)	0.169	0.73 (0.48–1.13)	0.16
Age					
13–15	284 (74.5)	1.00		1.00	
16–17	37 (63.8)	1.66 (0.93–2.98)	0.088	1.78 (0.96–3.28)	0.065
Ethnicity					
Malay	305 (72.3)	6.14 (0.81–46.80)	0.08	6.12 (0.80–47.12)	0.082
Others	16 (94.1)	1.00		1.00	
Type of school area					
Urban	143 (72.6)	1.00		1.00	
Rural	178 (73.6)	0.95 (0.62–1.46)	0.821	0.96 (0.61–1.49)	0.839
Father’s occupation					
Professional	152 (72.0)	1.00		1.00	
Non-professional	169 (74.1)	0.90 (0.59–1.37)	0.623	0.73 (0.42–1.25)	0.244
Mother’s occupation					
Professional	107 (71.3)	1.00		1.00	
Non-professional	214 (74.0)	0.87 (0.56–1.36)	0.543	0.86 (0.51–1.44)	0.569
Family monthly income					
≤RM 1500	37 (71.2)	1.00		1.00	
RM 1501–RM 2500	85 (72.6)	0.93 (0.45–1.92)	0.841	0.86 (0.408–1.797)	0.68
RM 2501–RM 5000	96 (73.3)	0.90 (0.44–1.84)	0.771	0.71 (0.32–1.57)	0.404
RM 5001–RM 8000	53 (73.6)	0.88 (0.40–1.96)	0.762	0.59 (0.228–1.519)	0.273
RM 8001–RM 15,000	36 (76.6)	0.75 (0.31–1.86)	0.54	0.50 (0.18–1.43)	0.195
≥PM 15,000	14 (70.0)	1.06 (0.34–3.27)	0.923	0.73 (0.21–2.52)	0.727

**Table 7:** Mean ± SD of knowledge, attitude and practices (KAP) and body composition.

	Knowledge	Attitude	Practice
**BMI**	*p* = 0.449	*p* = 0.198	*p* = 0.407
Underweight (*n* = 39)	50.26 ± 16.14	84.62 ± 24.48	40.51 ± 24.06
Normal (*n* = 245)	51.51 ± 16.59	87.76 ± 17.93	44.49 ± 23.42
Overweight (*n* = 81)	51.23 ± 14.70	91.60 ± 14.79	47.65 ± 20.34
Obese (*n* = 74)	51.46 ± 13.96	89.19 ± 18.19	43.51 ± 21.80
**Body Fat Percentage**	*p* = 0.992	*p* = 0.013 *	*p* = 0.381
Normal (*n* = 243)	51.85 ± 16.82	86.50 ± 19.64	43.70 ± 23.45
High (*n* = 196)	51.84 ± 14.45	90.81 ± 15.86	45.61 ± 21.70
**Waist circumference**	*p* = 0.419	*p* = 0.435	*p* = 0.243
Normal (*n* = 324)	51.48 ± 16.04	88.02 ± 18.88	45.30 ± 23.00
High (*n* = 115)	52.87 ± 15.09	89.57 ± 15.97	42.43 ± 21.71

* Significant difference between the mean (*p* < 0.05) by ANOVA test.
